# Multiple Sclerosis in the Asia Pacific Region: A Systematic Review of a Neglected Neurological Disease

**DOI:** 10.3389/fneur.2018.00432

**Published:** 2018-06-08

**Authors:** Wing L. Cheong, Devi Mohan, Narelle Warren, Daniel D. Reidpath

**Affiliations:** ^1^School of Pharmacy, Monash University Malaysia, Bandar Sunway, Malaysia; ^2^Jeffrey Cheah School of Medicine and Health Sciences (JCSMHS), Monash University Malaysia, Bandar Sunway, Malaysia; ^3^School of Social Sciences, Monash University, Clayton, Australia

**Keywords:** multiple sclerosis, epidemiology, Asia Pacific, prevalence, incidence, mortality, disability, registries

## Abstract

**Background:** Multiple sclerosis is thought to be relatively uncommon in the Asia Pacific region with prevalence estimated between 0 and 20 per 100,000. There is reason to doubt these estimates due to the lack of data from many countries and the growing evidence of variability in prevalence across small geographic areas. This study was conducted to systematically review the population prevalence, incidence, mortality and disability progression estimates of MS within the Asia Pacific region.

**Methods:** The systematic review was conducted on articles from 1985 till 31st July 2017 within the PubMed/MEDLINE, EMBASE, SCOPUS, and The Cochrane Library databases. The review included articles that were population-based studies conducted on patients with MS in the Asia Pacific region that reported either incidence, prevalence, mortality, or disease progression. Hospital-based studies and non-research articles were excluded to ensure that only information representative of the population was included for analysis. Data appraisal and extraction was done by independent reviewers. This review was registered with PROSPERO (ID: CRD42017082760).

**Findings:** Of the 2,757 articles found, 16 studies were included. Information on 6 (18.75%) of 32 Asia Pacific countries was found, with data representing 8% of the total population. Prevalence estimates were available for 6 countries while estimates for incidence (3 countries), mortality (4 countries), and disease progression (2 countries) were limited.

**Interpretation:** The lack of epidemiological data available in the Asia Pacific region creates a blind spot in the surveillance of MS which obscures the true burden of MS, causing patients to struggle to receive the resources and funding that they need.

## Introduction

Multiple sclerosis is one of the most common neurological diseases of the central nervous system with an estimated global prevalence in 2013 of 33 per 100,000. The global prevalence has increased from approximately 30 per 100,000 in 2008. The prevalence of multiple sclerosis, however, is not distributed equally across the globe. Studies have identified substantial variation in prevalence from Europe, as high as 100 per 100,000, to Africa, as low as 0.5 per 100,000 (1). Worldwide, women are more likely than men to suffer from multiple sclerosis in a ratio of 2 to 1 (2).

Multiple sclerosis is thought to be relatively uncommon in the Asia Pacific region. The Atlas of Multiple Sclerosis (Atlas of MS 2013), for instance, estimated the prevalence of MS in China, Korea, Taiwan, South East Asia and the Pacific between 0 and 5 per 100,000. The prevalence of MS in South Asia and Japan was estimated to lie between 5 and 20 per 100,000 (3). There is, however, good reason to doubt these estimates given the lack of data from many of the countries in the Asia Pacific region and the reliance on weak “expert opinion” for some of the data points (1, 3–5).

Neurological diseases such as multiple sclerosis impose a significant burden on low and middle-income countries. Improving the delivery of health care to patients with MS in each country would require an understanding of the local prevalence, distribution, and incidence of the disease as well as the effect that the disease has on the countries' disability and mortality profiles (6, 7). The lack of data is doubly important considering some evidence to suggest that the presentation and progression of MS in the Asia Pacific region may differ from the presentation and progression in Europe and North America (8, 9). Unfortunately, data collection systems and registries for neurological diseases that are necessary for understanding the burden of the disease are largely absent in the region (10).

A recent review of MS in the East, Southeast and South Asia was conducted by Eskandarieh and colleagues (11). The study focused on estimating the prevalence of MS and describing regional variation in the clinical features of MS. The review, unfortunately, gave only a passing nod to the largely insurmountable problem of reconciling estimates from population-based studies with those from hospital-based studies. As a consequence, their analysis is difficult to use for achieving even passable national or regional estimates. The study by Eskandarieh et al. also missed mortality and “time to disability” which are both critical outcomes in MS and worthy of consideration.

In this study we systematically reviewed the population prevalence and incidence estimates of MS, and also the mortality attributed to MS for each country and territory. We have included in the review the evidence on disease progression trends of MS as characterized by time to disability. We focused on countries and territories in the Asia Pacific region.

## Methods

### Search strategy

This review is a systematic review. The PubMed/MEDLINE, EMBASE, SCOPUS, and The Cochrane Library databases were searched for relevant articles published from 1985 till 31st July 2017.

In PubMed, the following search terms were used: (“Multiple Sclerosis”[MeSH]) AND (“Prevalence”[MeSH] OR “Morbidity”[MeSH] OR “Incidence”[MeSH] OR “Mortality” [MeSH] OR “Disease progression”[MeSH] OR “Epidemiology”[MeSH]OR “Expanded disability status score”[MeSH] OR “clinical course”[MeSH] OR “survival”[MeSH]) AND (“Country”[MeSH]).

After the initial search, a search alert was established to ensure subsequent relevant literature would not be excluded during the period of review. The reference lists of relevant articles were reviewed to identify additional relevant articles.

### Eligibility criteria

We included articles that met all of the following inclusion criteria: The articles were population-based studies (national or sub-national, i.e., state or districts) conducted on patients with multiple sclerosis; conducted in the Asia Pacific region, i.e., limited to any of the following countries: Bangladesh, Bhutan, Brunei, Cambodia, China, East Timor (or Timor Leste), Fiji, Hong Kong, India, Indonesia, Japan, North Korea, South Korea, Lao PDR (or Laos), Macau, Malaysia, Maldives, Mongolia, Myanmar (or Burma), Nepal, Pakistan, Papua New Guinea, Philippines, Samoa, Singapore, Solomon Island, Sri Lanka, Taiwan, Thailand, Tonga, Vietnam, or Vanuatu; reported one or more of the following data items: Data on incidence, prevalence, mortality (e.g., total number or cause-specific mortality rate), disease progression (e.g., mean age of onset of disease, time to disability milestones, mean age of death); cases of multiple sclerosis were diagnosed according to any of the following diagnostic criteria: Schumacher, Poser, or McDonald's criteria; and, in the case of “time to disability” studies, the Expanded Disability Status Scale was used for the “time to reach” milestones. No language limitations were imposed.

Hospital-based studies were excluded to ensure that only information representative of the population studied was used for analysis. Articles with abstracts only, conference proceedings, comments, editorials and letters to the editor were also excluded. The search was restricted to peer-reviewed literature with full-texts to ensure that only high-quality information was included for analysis.

### Data management and selection process

Retrieved articles were compiled into libraries within the EndNote reference management software and screened for duplicates. After the removal of duplicates, the list of article citations and abstracts were provided to two independent reviewers for inclusion or exclusion according to the criteria listed above. Choices for inclusion and exclusion were compared and any disagreements were discussed by the reviewers until a consensus was reached. The full texts of all articles selected for inclusion were provided to two reviewers for review and assessment. Whenever it was necessary, the authors of included articles were contacted for further information and/or additional data.

### Data extraction

A standardized data extraction form was developed to extract data from the reviewed articles. All eligible articles were reviewed by the two reviewers independently with the process being calibrated between them.

The information for extraction included the article's publication details (e.g., authors, year published); details of the study (e.g., country or location, and study period); epidemiological data (gender ratio, mean age of onset, prevalence, incidence, and mortality); and, disease progression data (time to disability milestones).

This protocol adhered to the Preferred Reporting Items for Systematic Reviews and Meta-Analysis Protocol 2015 (PRISMA-P) (12). This review was registered with PROSPERO (ID: CRD42017082760).

### Role of the funding source

There was no funding source for this study. The corresponding author had full access to all the data in the study and had final responsibility for the decision to submit for publication.

## Results

**Figure 1:**
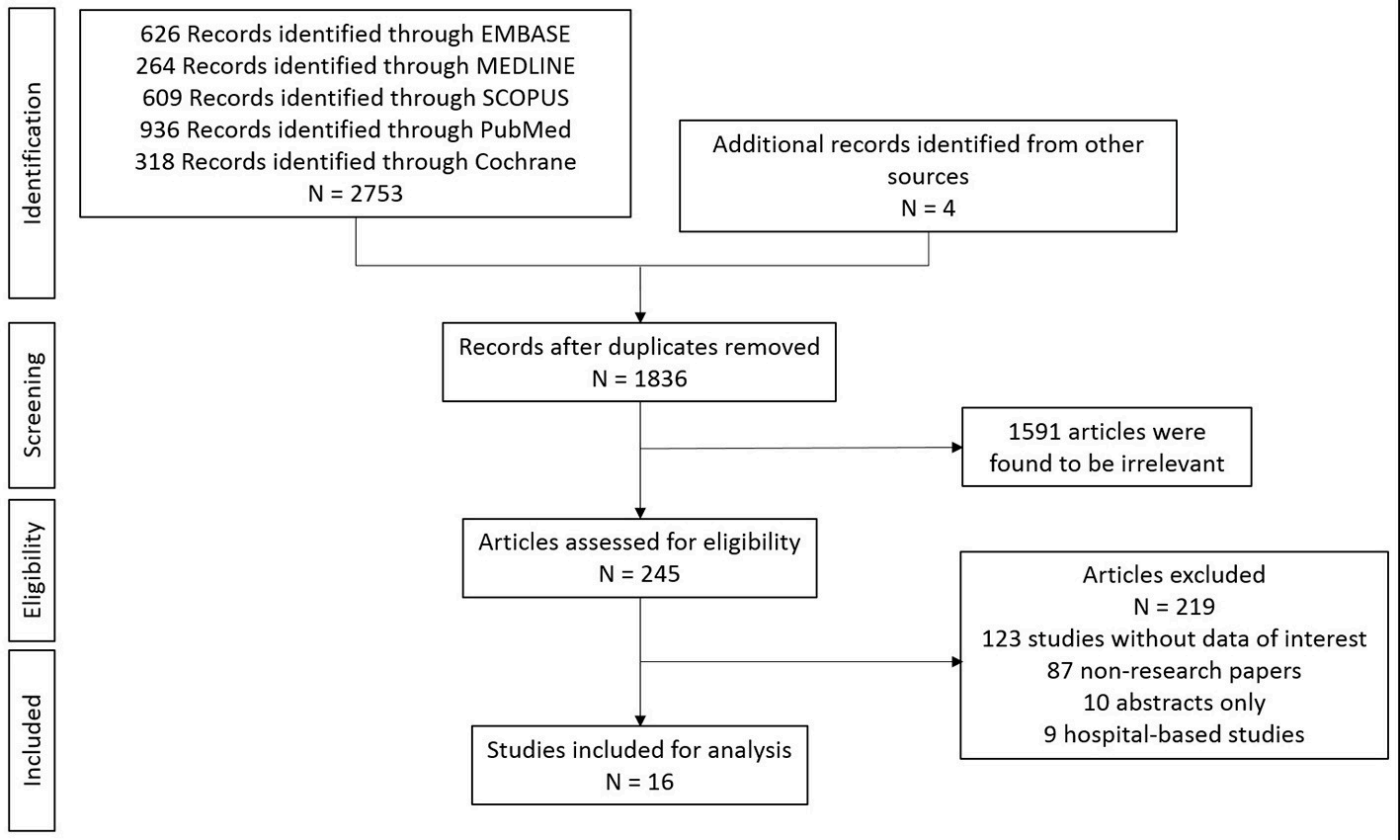
Article selection process of systematic review.

The initial search provided a list of 2,757 articles (2,753 from database search and 4 from reference lists of relevant articles). This was reduced to 1,836 articles after the removal of duplicates and further reduced to 245 articles after 1,591 articles were excluded due to irrelevance on the basis of their titles and abstracts. 245 articles were reviewed in-depth and after the removal of ineligible studies, 16 studies were selected for inclusion in this review (see Figure 1).

Only 6 of the 32 countries included in the geographical region had published literature on the epidemiology of multiple sclerosis, namely: China, Hong Kong, India, Japan, South Korea, and Taiwan (see Table 1).

**Table 1:** Epidemiological data of multiple sclerosis in the Asia Pacific region (^*^calculated based on the data reported in the study).

**Study**	**Study period**	**Location**	**Size of studied population**	**Gender ratio (F:M)**	**Age of onset**	**Prevalence (per 100,000)**	**Incidence**	**Mortality**	**Disease progression**
**JAPAN (6 ARTICLES)**									
Araki et al. 13	1 Jan 1977–30 Jun 1982	Japan/Kumamoto city	1.8 million	6:1	36.6 (range 14–54; mean)	1.3	N/A	N/A	N/A
Houzen et al. 14	2001	Japan/Hokkaido/Tokachi Province	361,726	2.9:1	29.1 ± 14.2 (range 7–60)	8.57 (95% CI: 5.82-12.17)	N/A	N/A	N/A
Itoh et al. 15	2002	Japan/Asahikawa	363,526	1.8:1	RRMS: 29.0 ± 9.5 SPMS: 38.6 ± 6.8 PPMS: 37.8 ± 6.6	10.2 (95% CI: 7.17–14.03)	N/A	N/A	N/A
Kira et al. 8	1 Jan−31 Dec 2003	Japan	127 million	2.9:1	32 ± 13 (mean)	7.7 (95% CI: 7.1–8.4)	N/A	N/A	N/A
Houzen et al. 16	(1) 2001–2006: Changes in MS prevalence over the last 10 years (Prevalence day 31 Mar 2006) (2) 1975–2004: Changes in MS prevalence and incidence	Japan/Hokkaido/Tokachi Province	361,726	1974: 1.7:1 2001: 2.9:1 2006: 2.9:1	2001: 29.1 ± 14.2 (range 7–60) 2006: 28.4 ± 11.7 (range7–60)	2001: 8.57 (95% CI: 5.82–12.17) 2006: 13.1 (95% CI: 9.6–17.4)	Average annual incidence rate per 100,000: 1975–1979: 0.06 (95% CI: 0.02–0.33) 1980–1984: 0.17 (95% CI: 0.04–0.50) 1985–1989: 0.23 (95% CI: 0.06–0.60) 1990–1994: 0.5 (95% CI: 0.23–0.95) 1995–1999: 0.78 (95% CI: 0.43–1.31) 2000–2004: 0.78 (95% CI: 0.43–1.31)	N/A	N/A
Houzen et al. 17	(1) 2001–2011: Changes in MS prevalence over the last 10 years (Prevalence day 31 Mar 2011) (2) 1975–2011: Incidence of MS over the last 30 years	Japan/Hokkaido/Tokachi Province	361,726	2001: 2.63 2006: 2.75 2011: 3.38	2001: 28.8 ± 13.5 2006: 28.4 ± 11.8 2011: 28.3 ± 11.2	2001: 8.1 (95% CI: 5.4–11.7) 2006: 12.6 (95% CI: 9.2–16.9) 2011: 16.2 (95% CI: 12.3–21.1)	Average annual age- and sex-adjusted incidence rate for 10-year intervals per 100,000: 1980–1989: 0.17 1990–1999: 0.58 2000–2009: 0.77	N/A	N/A
**CHINA (3 ARTICLES)**									
Cheng et al. 18	1 Sep 2004–31 Aug 2005. Prevalence date: 31 Dec 2004	China/Shanghai	8.86 million	1.8:1	39.8 ± 14.2 (mean)	1.39 (95% CI: 1.16–1.66)	N/A	N/A	N/A
Cheng et al. 19	1 Sep 2004–31 Aug 2005	China/Shanghai	8.86 million	1.4:1	37.4 ± 14.2 (mean)	N/A	N/A	N/A	Time to EDSS 3: 6.62 years; Time to EDSS 7: 8.31 years
Liu et al. 20	2013	China/Shandong province	95.8 million	N/A	N/A	DISMODII software estimate: 3.7 (95% CI: 1.65–5.8) (male), 6.7 (95% CI: 2.7–9.56) (female)	DISMODII software estimate: 0.12/100,000 (male), 0.2/100,000 (female)	DISMODII software estimate: 21 deaths (male), 32 deaths (female)	N/A
**HONG KONG (2 ARTICLES)**									
Yu et al. 21	1 Jan 1981–31 Dec 1987	Hong Kong	5.65 million	1.8:1	29 ± 12 (range 13–59)	0.88	N/A	9% (Number of death: 4)	N/A
Lau et al. 22	Jan–Jun 1999	Hong Kong	6.8 million	9.6:1	29.4 (range 11–60; mean)	0.77	N/A	N/A	N/A
**TAIWAN (3 ARTICLES)**									
Tsai et al. 23	Jan 1985–Dec 1999	Taiwan	22.4 million	5:1	29.93 (range 11–70; mean)	1.9	N/A	9.3%	N/A
Lai and Tseng 24	2000–2005	Taiwan	22.3–22.8 million	2.5:1	N/A	2000: 1.68 2001: 2.02 2002: 2.18 2003: 2.47 2004: 2.80 2005: 2.96	Average annual incidence rate per 100,000: 2001: 0.81 2002: 0.80 2003: 0.82 2004: 0.83 2005: 0.67	N/A	N/A
Tsai and Lee 25	1 Jan 1997–31 Dec 2009	Taiwan	23.1 million	3.3:1	N/A	2009: 4.99^*^	Average annual incidence rate per 100,000: 1997–2008: 0.448^*^	88 number of death; 7.1%; SMR 7	Mean survival (EDSS 10) 116.92 months (95% CI: 114.61–119.23)
**SOUTH KOREA (1 ARTICLE)**									
Kim et al. 26	2000–2005, 6 years	South Korea	47 million	1.26:1	38.3 ± 14.1	3.5 (95% CI: 3.0–3.8)	N/A	152 deaths (1990–2003)	N/A
**INDIA (1 ARTICLE)**									
Pandit and Kundapur 27	January 2011–June 2013	India/Karnataka/(urban) Mangalore	419,306	1.69:1	38.3 ± 12.8 (mean)	8.3	N/A	N/A	N/A

### Japan

Six articles from Japan were included in this review. The Japanese studies identified in this review included one nationwide survey, as well as a series of sub-national studies in Kumamoto district, Tokachi province, and Asahikawa city. Three studies published in 2003, 2008, and 2012 reported the prevalence of MS in Tokachi province. In 2001, the prevalence of MS in Tokachi province was estimated at 8.57 per 100,000 (95% CI: 5.82–12.17) (14). In 2006, Houzen et al estimated the prevalence at 13.1 per 100,000 (95% CI: 9.6–17.4) and this was later reevaluated to be at 12.6 per 100,000 (95% CI: 9.2–16.9) (16, 17) In later studies, the prevalence of MS in Tokachi province was estimated at 16.2 per 100,000 in 2011 (95% CI: 12.3–21.1) (17). The prevalence reported in these studies suggest the possibility of increasing prevalence. There was insufficient information provided to determine if the increases in prevalence were statistically significant. There was significant increase reported in the incidence of MS in Tokachi province. The annual incidence per 100,000 population increased from 0.06 (95% CI: 0.02–0.33) for the period between 1975 and 1979, to 0.78 (95% CI: 0.43–1.31) for the period between 2000 and 2004 (16). Using different study periods, Houzen et al later estimated the incidence of MS in Tokachi province at 0.17 per 100,000 population (1980–1989), 0.58 per 100,000 (1990–1999), and 0.77 per 100,000 (2000–2009) (17). Houzen et al. speculate that the increase in the prevalence of MS in Tokachi province may be related to the reported increase in incidence as opposed to changes in diagnostic capabilities, which did not change during the study period. The reasons behind the increased incidence, however, remain unclear (16, 17). The studies in Kumamoto district and Asahikawa city estimated the prevalence of MS at 1.3 per 100,000 and 10.2 per 100,000 (95% CI: 7.17–14.03), respectively, indicating variances in the prevalence of MS across Japan (13, 15). The nationwide survey conducted in 2004 estimated the prevalence of MS in Japan at 7.7 per 100,000 (95% CI: 7.1–8.4). This study, however, did not report on the incidence or mortality of MS in Japan (8). None of the Japanese studies reported data on time to disability milestones or mortality figures.

### China

Three articles from China were included in this review. There were no national studies identified. The studies conducted in China include two sub-national studies, one conducted in Shanghai and one study in Shandong province. Cheng et al estimated the prevalence of MS in Shanghai on 31st December 2004 at 1.39 per 100,000 (95% CI: 1.16–1.66) (18). In a subsequent study on the same patient population in Shanghai, Cheng et al. reported that the mean time taken to reach EDSS 3 (Moderate disability in one functional system, or mild disability in three or four functional systems with no impairment to walking) and EDSS 7 (Unable to walk beyond approximately 5 m even with aid. Essentially restricted to wheelchair; though wheels self in standard wheelchair and transfers alone. Up and about in wheelchair some 12 h a day) was 6.62 and 8.31 years, respectively (19). Liu et al, using DISMOD II modeling, estimated the prevalence of MS in the Shandong province in 2013 at 5.2 per 100,000; 3.7 per 100,000 (95% CI: 1.65–5.8) for males and 6.7 per 100,000 (95% CI: 2.7–9.56) for females. Incidence was statistically estimated in Shandong at 0.12 per 100,000 population per year (males) and 0.2 per 100,000 population per year (females) while the number of MS deaths in Shandong were estimated at 53 deaths in 2013 (20). There were no other studies reporting on incidence and mortality of MS in China.

### Hong Kong

Two articles from Hong Kong were included in this review. Both articles reviewed provided territory-wide estimates of MS prevalence in Hong Kong. Yu et al estimated the prevalence of MS in Hong Kong at 0.88 per 100,000 on 31st December 1987 while Lau et al estimated the prevalence at 0.77 per 100,000 in 1999 (21, 22). In the past 17 years, there have been no new territory-wide studies. Incidence and disease progression data for Hong Kong was not available in the studies reviewed while mortality data was reported in only one study; Yu et al reported 4 deaths from 1981 to 1987 (total cases: 47).

### Taiwan

Three articles from Taiwan were included in this review. There were two studies using data from Taiwan's National Health Insurance database which reported the prevalence of MS in Taiwan. The results from these studies indicate an increasing trend in the prevalence of MS: 1.9 per 100,000 in 2002 and increasing to 2.96 per 100,000 in 2005 (23, 24). Lai et al. estimated the annual incidence of MS at 0.8–0.83 per 100,000 between 2001 and 2004, while in 2005, the incidence decreased to 0.67 per 100,000 (24). Separately, using the data reported by Tsai et al. we estimated the prevalence of MS at 4.99 per 100,000 in 2009 and the annual incidence from 1997 to 2008 at 0.448 per 100,000 (25). The reasons for the increase in prevalence from 2002 to 2009 is unclear, considering the fact that incidence has not increased and diagnostic capabilities during this time period were unchanged. Disease progression timelines were reported by Tsai et al. where the mean survival time or mean time to death (EDSS 10) was reported at 116.92 months (95% CI 114.61–119.23) (25). Mortality was reported in two studies. Tsai et al reported a crude mortality rate of 9.3% from the study population of 43 patients during the study period of 1985–1999 (23). In another study, Tsai et al estimated the Standardized Mortality Ratio of 7 and a mortality rate of 7.1% for the study period from 1997 to 2008 (25).

### South Korea

One article from South Korea was included in this review. Kim et al. used the National Health Insurance database from 2000 to 2005 to estimate the national prevalence of MS in South Korea −3.5 per 100,000 (95% CI: 3.0–3.8). The study also identified 152 MS deaths between 1990 and 2003 (26). There was no information reported on the incidence of MS in Korea.

### India

One article from India was included in this review. In 2013, the registry of MS patients in the city of Mangalore was analyzed and the prevalence was estimated at 8.3 per 100,000 (27). There were no nationwide prevalence or epidemiological studies for India. There were also no studies reporting the incidence, mortality or disease progression of MS in India in this review.

Each of the articles reviewed here provides estimates of MS prevalence, incidence, or mortality at a national or sub-national level. Using 2016 World Bank population data we examined the proportion of the total regional and national populations represented by those studies (https://data.worldbank.org/indicator/SP.POP.TOTL). The total population of the geographic areas covered by the research articles' sampling strategies was 0.316 Billion people. This number is less than the population of the countries' in which the studies were conducted because many of the estimates are based on sub-national samples. The total population of the countries in which the studies were based was 2.93 Billion. In other words, the studies' sampling strategies represented about 11% of the total population of the countries involved. Many of the countries in the region, however, had no studies—representing about 1.087 Billion people. Thus, the studies' sampling strategies represented about 8% of the total regional population of the region. Alternatively, there are no population estimates of MS for 92% of the regional population; i.e., 3.7 Billion people. Figure 3 shows the geographical areas at a sub-national level, for which representative MS estimates exist.

## Discussion

With the scarcity of information, the lack of surveillance systems, and the limited resources devoted toward treating it, multiple sclerosis is the neurological equivalent of a neglected tropical disease. There is an evident lack of epidemiological data available in the Asia Pacific region, with only 6 out of the 32 countries and territories searched having any published studies at all. In terms of population coverage, the region studied in this review has an estimated population of 4 billion while the studies included in this review provided data over a combined geographic area that has an estimated population of 317 million. As such, there are estimates available for only about 8% of the population in the region. This creates a persistent blind spot in the global surveillance of MS.

The primary source of the estimates reported in the reviewed articles were mainly from population surveys and national health insurance databases (for the studies conducted in Taiwan and Korea). The modeled estimates by Liu et al. were also based on government reported health statistics. The methods and the primary sources of data used in these studies provides confidence in the reported estimates. The high-cost and the surveillance infrastructure required (in the case of having a national health insurance database) to conduct similar studies may partially explain the lack of such studies in the region, especially among the lower-income countries.

**Figure 2:**
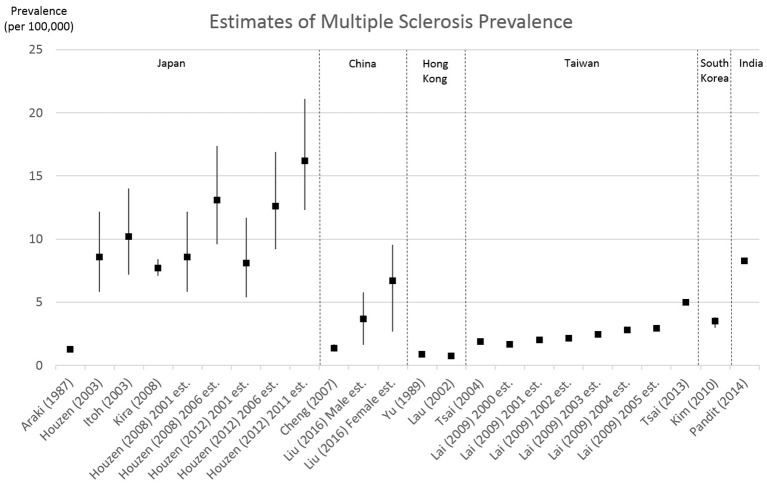
Estimates of multiple sclerosis prevalence from the articles reviewed. Bars represent reported 95% confidence intervals.

While the prevalence data that was available in the studies reviewed generally matches the broad categorical estimates made in the Atlas of MS 2013, assuming that one can extrapolate from studies drawn from a population of 317 million people to another 3.7 billion people completely outside the sampling strategy would stretch credulity. Recent studies have also indicated that prevalence is increasing in several countries (28). This was also observed in the studies conducted in Hong Kong, Taiwan, and Japan identified in this review (see Figure 2). Without local surveillance systems for MS it is unclear if other countries are similarly facing an increasing trend in MS prevalence.

**Figure 3:**
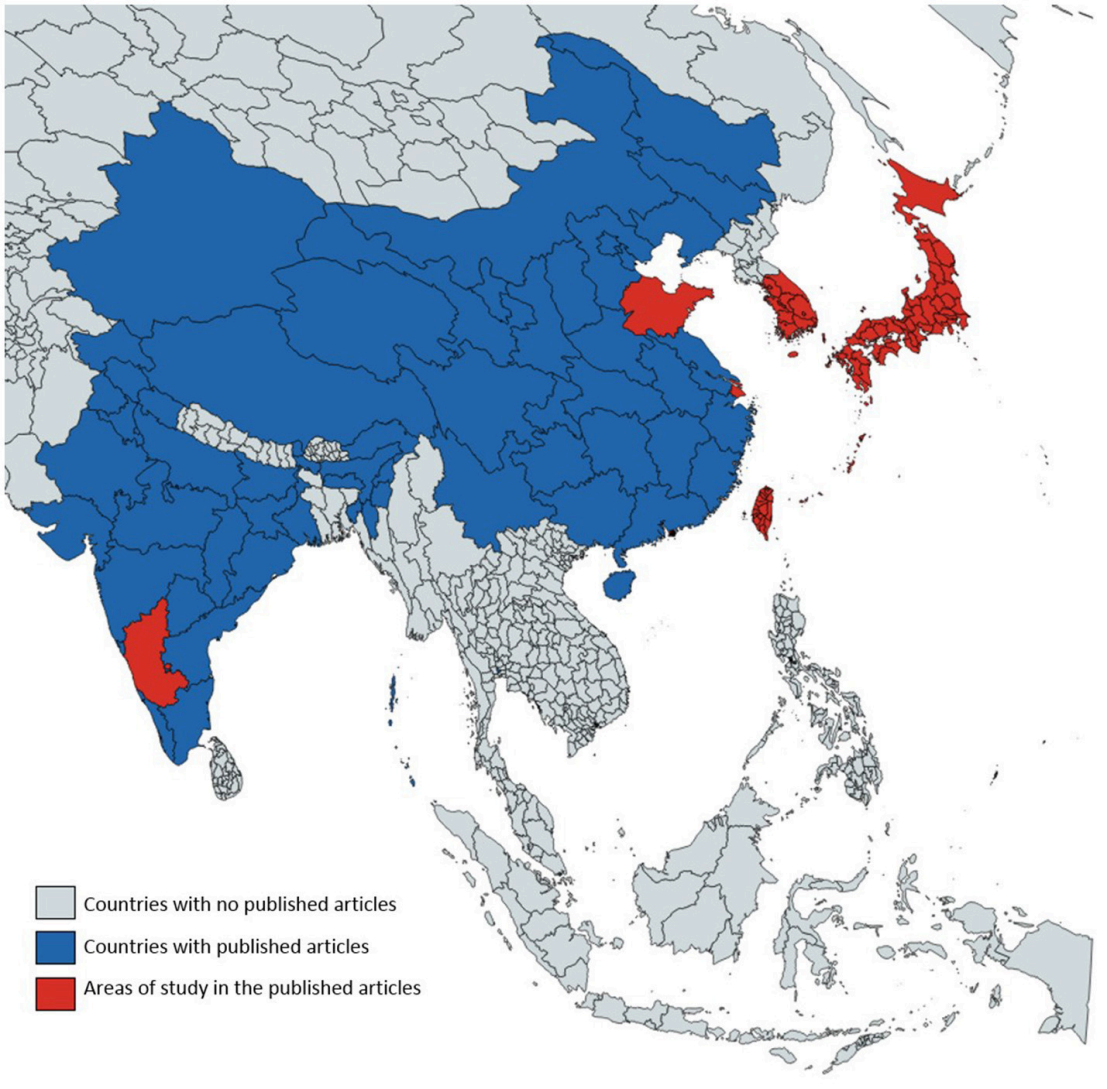
Geographical areas with existing published estimates on MS epidemiology.

The Atlas of MS 2013 made no estimates of incidence, mortality and disability timelines of MS in the Asia Pacific region which means that countries without their own data have no regional estimate to fall back on—however inadequate these may be (3). The older Atlas of MS 2008 estimated the incidence of MS in the Western Pacific region at 0.9 per 100,000 (4). The studies in this review reported incidence rates for this region that were similar to the Atlas of MS 2008 estimate—however, this is not altogether surprising because they draw on similar data sources. An unpublished abstract presented in 2015 however, estimated the MS incidence rate for Hong Kong at 2.2 per 100,000 (29). No data was reported for Southeast Asia and this review did not find any published data for the region either (4). There were no regional mortality estimates of MS while the Global Burden of Disease Study 2015 reported the age-standardized rate (per 100,000) of deaths due to MS as zero (6). Unsurprisingly, this review found only limited descriptions of MS mortality in the region. A similar lack of data about disability trends in the region was also noted.

These findings are in line with the observation made in the Atlas of Country Resources for Neurological Disorders regarding the lack of reporting systems for neurological disorders and data collection systems throughout the African, South East Asia and Western Pacific Regions (10).

Several implications arise from the lack of epidemiological data and poor surveillance systems in so many countries within the Asia Pacific region.

Without local national and sub-national-level data for each country, policy makers and governments rely on the estimates made in reports like the Atlas of MS 2013. The methodology (possibly the best available) is seriously deficient, and has important consequences for national decisions about resource allocation and service delivery (30). While policy makers and governments may need to rely on regional estimates of prevalence in the absence of national data, the same cannot be done for measures of incidence, mortality and disability trends, because they simply do not exist. There is, furthermore, good reason to believe that a reliance on regional estimates is at best a stop-gap measure on the path to each country developing its own adequate surveillance systems. This lack of comprehensive and up-to-date data raises the significant question of whether MS has been neglected in these countries and territories—following the anecdotal observation that if we cannot count it, it does not matter.

To be clear, there is certainly no lack of research on MS within the region; there is an important body of clinical and therapeutic research being done on MS by researchers in China, Japan, Taiwan, Korea and other Asian countries. The lack of published epidemiological research in the majority of the countries and territories reviewed, however, obscures the true burden of MS in this region. Many of the countries and territories studied in this review are low and middle-income countries where resources for dealing with neurological diseases are disproportionately scarce (10). If the disease burden of MS in these countries is underestimated, or worse, unknown, then MS patients will inevitably struggle to receive the resources and funding that they need (7). It is impossible to provide resources for a problem that (as a matter of faith) you do not believe exists.

The challenge faced by the neglected neurological diseases (NNDs) is paralleled by the now well recognized problem of the neglected tropical diseases (NTDs). The efforts to treat NTDs are frequently stymied by the absence of baseline information on prevalence and disease burden in the scientific literature as well as the lack of surveillance efforts. It is well-established that the treatment of neglected diseases will require epidemiological and demographic information that can only be obtained through the establishment of disease monitoring and evaluation systems that policy makers and researchers can analyze to determine the burden of disease on health systems and the impact of control initiatives (31–34). While MS might not be a “Neglected Tropical Disease” in the exact sense of the term, this review shows that it might very well be a “Disease Neglected in the Tropics.”

## Author contributions

WC and DR carried out the study, collected the data and edited the manuscript. NW and DM provided input for the search strategy, analysis and edited the manuscript. All authors contributed to study design, analysis of data, and the initial manuscript write-up.

### Conflict of interest statement

The authors declare that the research was conducted in the absence of any commercial or financial relationships that could be construed as a potential conflict of interest.
